# Cold and heavy: grasping the temperature–weight illusion

**DOI:** 10.1007/s00221-020-05794-y

**Published:** 2020-03-27

**Authors:** Johann P. Kuhtz-Buschbeck, Johanna Hagenkamp

**Affiliations:** Institute of Physiology, Christian-Albrechts-University, Hermann-Rodewald-Str. 5, 24118 Kiel, Germany

**Keywords:** Temperature–weight illusion, Object lifting, Weight perception, Grip force

## Abstract

The apparent heaviness of weights placed on the skin depends on their temperature. We studied the effects of such a temperature–weight illusion (TWI) on perception and action in 21 healthy volunteers. Cold (18 °C), thermal-neutral (32 °C, skin temperature) and warm (41 °C) test objects were placed onto the palm of the non-dominant hand. Their veridical mass was 350 g (light) or 700 g (heavy). Perception of heaviness was assessed with two psychophysical experiments (magnitude estimation, cross modal matching). Cold heavy objects felt about 20% heavier than thermal-neutral objects of the same mass, shape and material. In a subsequent grip-lift experiment, the test objects were grasped with a precision grip of the dominant hand and lifted off the palm of the non-dominant hand. The grip and lift forces exerted by the fingertips were recorded. The temperature of the objects had significant effects (ANOVA, *p* < 0.05) on the peak grip and lift forces and on the peak grip force rate (i.e., the initial force incline). The peak grip force was about 10% higher when cold heavy objects were grasped and lifted, compared to lifts of otherwise identical thermal-neutral objects. The TWI was less pronounced when light objects or warm objects were handled. In conclusion, cooling of an object increases its apparent heaviness (perception) and influences scaling of the fingertip forces during grasping and lifting (action).

**Electronic supplementary material:**

The online version of this article (10.1007/s00221-020-05794-y) contains supplementary material, which is available to authorized users.

## Introduction

A cold coin placed on the forehead of a supine person feels heavier than a coin at room temperature. This temperature–weight illusion (TWI) was originally described with a silver thaler by Ernst Heinrich Weber in 1846 (translated by Ross and Murray 1996). More than 100 years later, Stevens and colleagues explored the TWI in a series of experiments (Stevens and Green 1978; Stevens 1979). They let healthy volunteers judge the apparent heaviness of cold (0 °C), neutral (32 °C), and warm (45 °C) aluminum disks, which were placed on the skin of the hand, forearm, and other body regions. The disks, the actual mass of which ranged between 21 and 105 g, were not lifted actively so that only thermal and tactile (skin deformation) cues to their heaviness were available. Stevens found that in particular cold light disks felt considerably heavier than neutral, identically weighted disks. Warmth intensified the perceived heaviness, too, but to a lesser extent. The possibility cannot be excluded, however, that Steven’s warm and cold stimuli induced discomfort or pain in some subjects, which might have influenced the results. Quantitative sensory testing of a large control group (Rolke et al. 2006) has shown that the mean heat-pain threshold of the skin is at ~ 44 °C, and that cold pain begins at about ~ 14 °C when the skin temperature is gradually lowered with a thermode.

When an object is grasped and lifted, our expectation of how heavy it will be allows us to scale the grip and lift forces predictively, without having to rely on time-consuming feedback (Johannson 1996). Unless the object being lifted is familiar, motor planning is based on the expected weight, the surface properties and shape of the item. People have strong experience-dependent expectations (motor priors) about object weight, which are supported by consistent patterns in the environment (Buckingham 2014). Objects made of dense material such as steel are expected to be heavier than equally sized objects made of less dense material such as styrofoam (Baugh et al. 2012; Buckingham et al. 2011), and a positive correlation between volume and weight is expected (Flanagan and Beltzner 2000; Buckingham and Goodale 2010). The effects of such expectations on action and perception have been investigated with other scenarios [material–weight-illusion, size–weight-illusion, see Buckingham (2014) for review]. However, it is not yet known if cold objects are expected to be heavier than thermal-neutral objects of the same size when they are grasped and lifted. The TWI described by Weber and Stevens implies that such a motor prior may exist, although an illusion can have different effects on perception and action.

The present study of normal volunteers aims (i) to find out whether a TWI can be elicited with innocuous thermal stimuli (cold = 18, neutral = 32, warm = 41 °C) and (ii) to test whether cold objects are grasped and lifted with higher grip force than otherwise identical thermal-neutral objects. The same applies to warm objects. The test objects, which had two different actual weights (700 g, 350 g), were placed onto the palm of the non-dominant hand. First the participants judged their apparent heaviness in two psychophysical experiments, and then they grasped and lifted the objects off the palm with a precision grip of the dominant hand. Since the initial grip and lift forces are scaled predictively according to the expected weight (Gordon et al. 1993; Nowak et al. 2013), we hypothesized that higher peak forces would be applied to the cold (and warm) objects, the apparent heaviness of which may be augmented by the TWI. We included experimental conditions with, and conditions without proprioceptive sensory information about the actual weight of each object.

## Methods

Twenty-one healthy volunteers (ten women, eleven men) with a mean age of 24 ± 3.5 years (SD, standard deviation) participated in the study. Most of them were university students and all were naïve to the specific purpose of the experiments, which had been approved by the Ethics Committee of the Medical Faculty of the University of Kiel (D 442/19). Each participant gave written informed consent. Nineteen participants were right-handed and two were left-handed according to a questionnaire (Oldfield 1971). The volunteers were told that they should first judge the heaviness of objects of different weight and temperature, and later grasp and lift these objects. All tests were initially explained and rehearsed with practice objects (which were not used later on). The temperature–weight illusion was not explicitly mentioned or demonstrated until the very end of the experiments, which lasted about two hours.

### Judgment of apparent heaviness

**Fig. 1:**
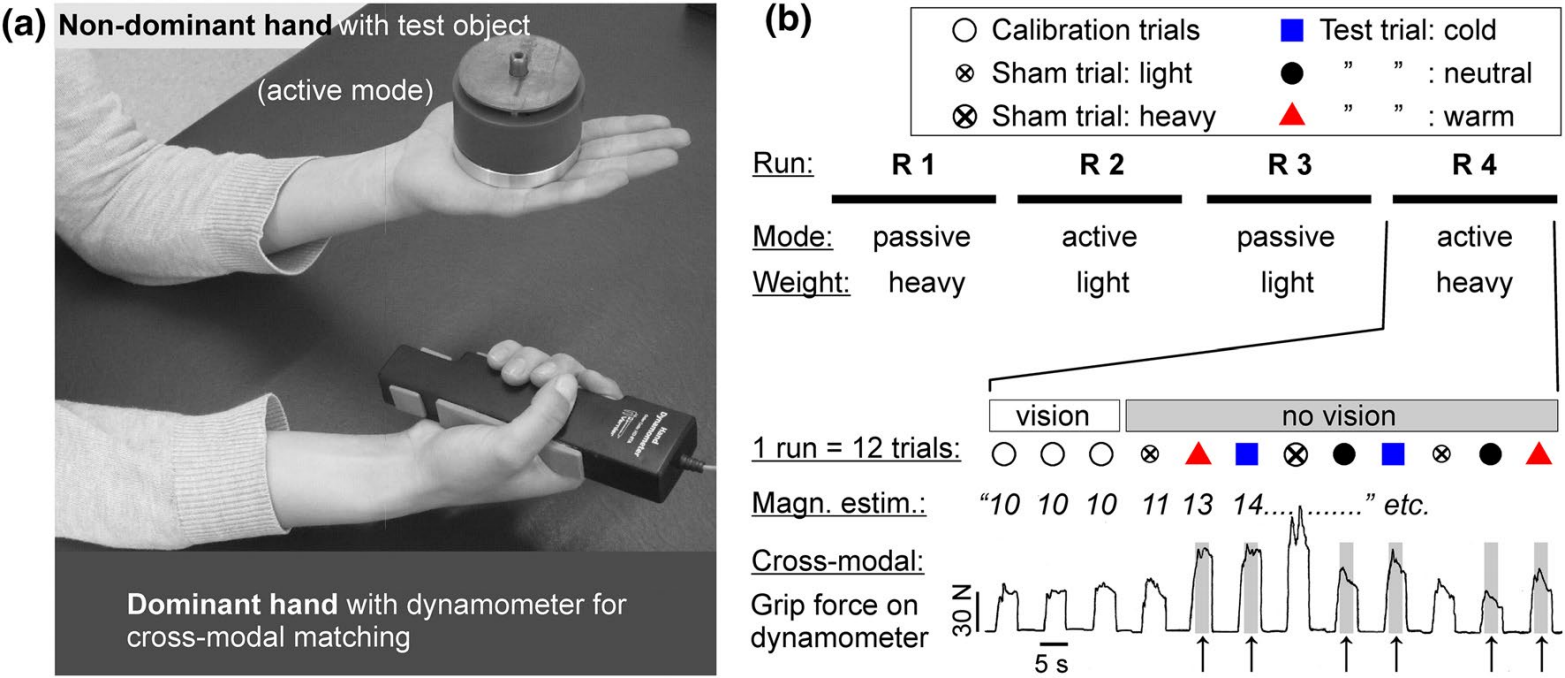
**a**, **b** Psychophysical experiments. **a** A test object was placed on the palm of the non-dominant hand. The dominant hand held a 500-g standard object during the numerical ratings of heaviness (1st experiment, magnitude estimation), or, as shown here, squeezed a dynamometer during cross-modal matching (2nd experiment). **b** Each experiment consisted of four runs (R 1–4) with 12 trials each. Mode of handling, veridical object weight and the sequence of trials varied in pseudorandomized order (see supplementary data). Arrows denote the force pulses applied on the dynamometer during cross-modal matching when the non-dominant hand carried objects of different temperature (cold = 18, neutral = 32, warm = 41 °C) but identical mass

In two psychophysical experiments (magnitude estimation, cross-modal matching), participants judged the apparent heaviness of thermal-neutral (32 °C), cold (18 °C), and warm (41 °C) objects. They sat on an adjustable chair in front of a padded table. Just before each trial, an assistant passed one object under a curtain to the experimenter, who placed it onto the palm of the participant’s non-dominant flat hand. In the active mode, the participants held the object ~ 10 cm above the table surface, with their elbow propped on the table and their forearm supinated (Fig. 1a). In the passive mode, the non-dominant hand rested on a small cushion on the table (palm facing upward). Participants were told to relax their arm and hand muscles and to just sense the object’s pressure on their palm in this mode so that no proprioceptive information (muscle tension, force) about its weight was available. In addition to the object's temperature and mode of handling (active, passive), the objects’ mass was varied, too (see below).

**Fig. 2:**
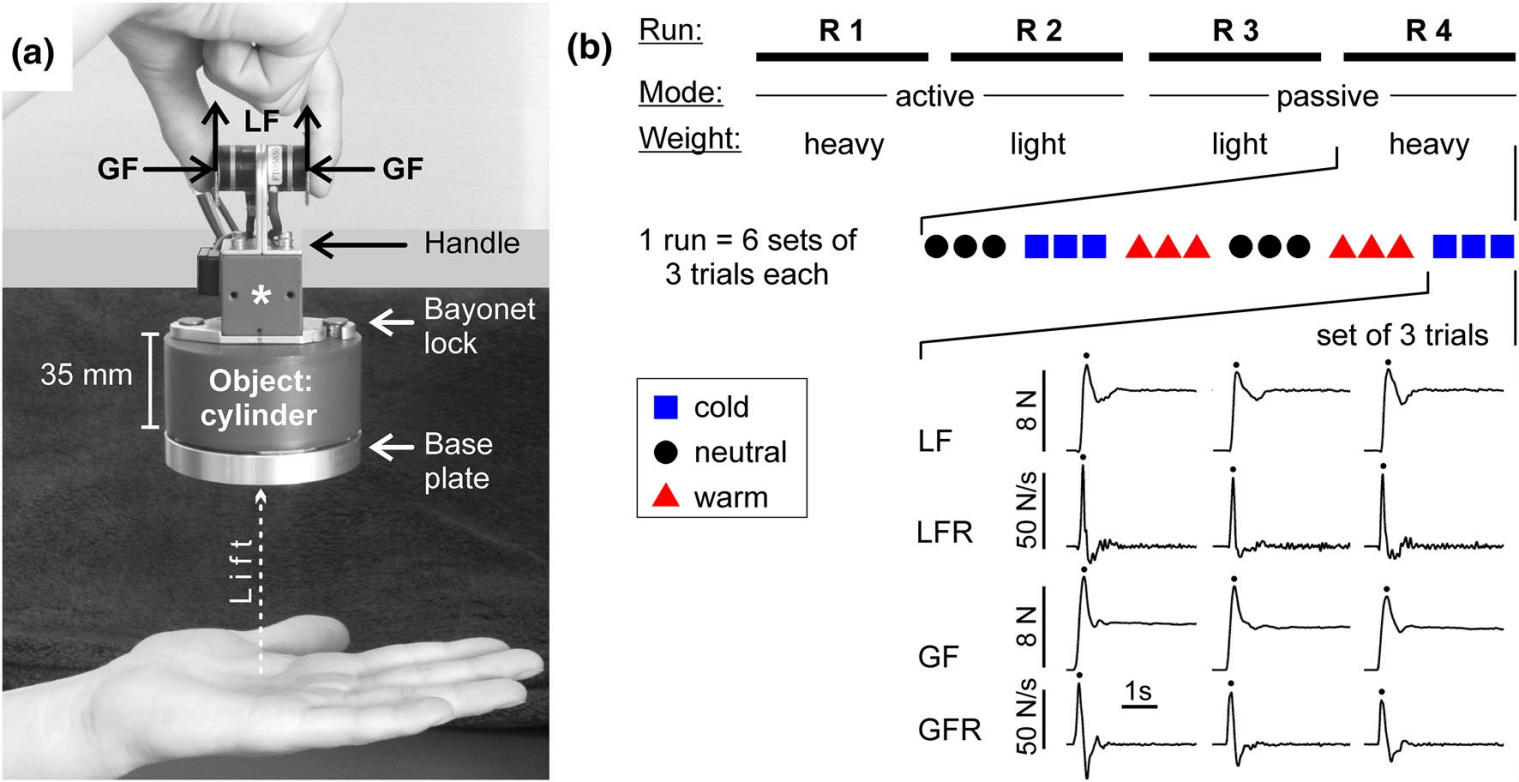
**a**, **b** Grip-lift experiment. **a** The test object was lifted from the palm of the non-dominant hand with a precision grip of the other hand. Transducers attached to a handle (asterisk) measured the grip (GF) and lift forces (LF) of the thumb and index finger. **b** The grip-lift experiment consisted of four runs (R 1–4) with 18 trials each, namely six sets of three consecutive lifts of one test object with a given temperature (e.g. 32 °C; see supplementary data concerning randomization). Typical lift and grip force curves (LF, GF) and their first derivatives (LFR, GFR) are shown; their maxima are marked with dots

The test objects were six identical-looking hollow plastic cylinders (diameter 75 mm, height 35 mm), each with a 15 mm-thick massive aluminum base plate. The bottom of this plate was slightly curved so that it fit snugly into the palm of a flat hand. The plastic cylinders were partly filled with lead disks that were fastened to a central threaded rod (see supplementary Fig. 1). A further disk placed onto the object accounted for the mass of the force transducers used in the later grip-lift experiment (compare Figs. 1a and 2a). The total weight was either 350 g (light) or 700 g (heavy). The test objects were brought to the preset desired temperatures with thermostatically controlled cool boxes, a warming cabinet (Memmert U 100, Schwabach, Germany), and heating plates, which were hidden from the participants behind curtains. Objects were taken out of the boxes/cabinet just before each trial, and their temperature was checked regularly with a contact thermometer (Testo Mini, Lenzkirch, Germany). Two further objects weighing 500 g served as standard stimuli in calibration trials. They were of the same shape as the test objects and had neutral temperature (32 °C).

Each psychophysical experiment consisted of four runs with short pauses in between (Fig. 1b). The mode of handling (active, passive) and actual test object weight (350 g, 700 g) varied in randomized order between runs. Each run consisted of 12 successive trials. Three initial calibration trials were performed under full vision with the 500 g standard object. Participants were blindfolded during the following nine trials. Six of these trials were performed with test objects of different temperatures (18, 32, 41 °C, i.e. cold, neutral, warm), whose actual weight remained constant (350 g or 700 g) within each run. Three sham trials were interspersed (Fig. 1b), two with a light and one with a heavy “dummy” object. These sham trials ensured that the participants, unaware of our hypothesis, experienced obvious variations of the object weight within each run, and did not conjecture that they were to be “tricked” by a temperature–weight illusion while the veridical mass remained constant. Dummies used for sham trials were brass weights (temperature 32 °C) that were 43% lighter or 43% heavier than the current test object. Hence, they weighed 200 g/500 g in experimental runs with light (350 g) test objects, and 400 g/1000 g in runs with heavy (700 g) test objects. The order of trials was pseudorandomized (see supplementary Fig. 2) to avoid potential systematic aftereffects of the changes in mass (Cole et al. 2008; Nowak et al. 2013); e.g., cold test objects were not regularly preceded by sham trials with light dummies.

#### Magnitude estimation of apparent heaviness

**Fig. 3:**
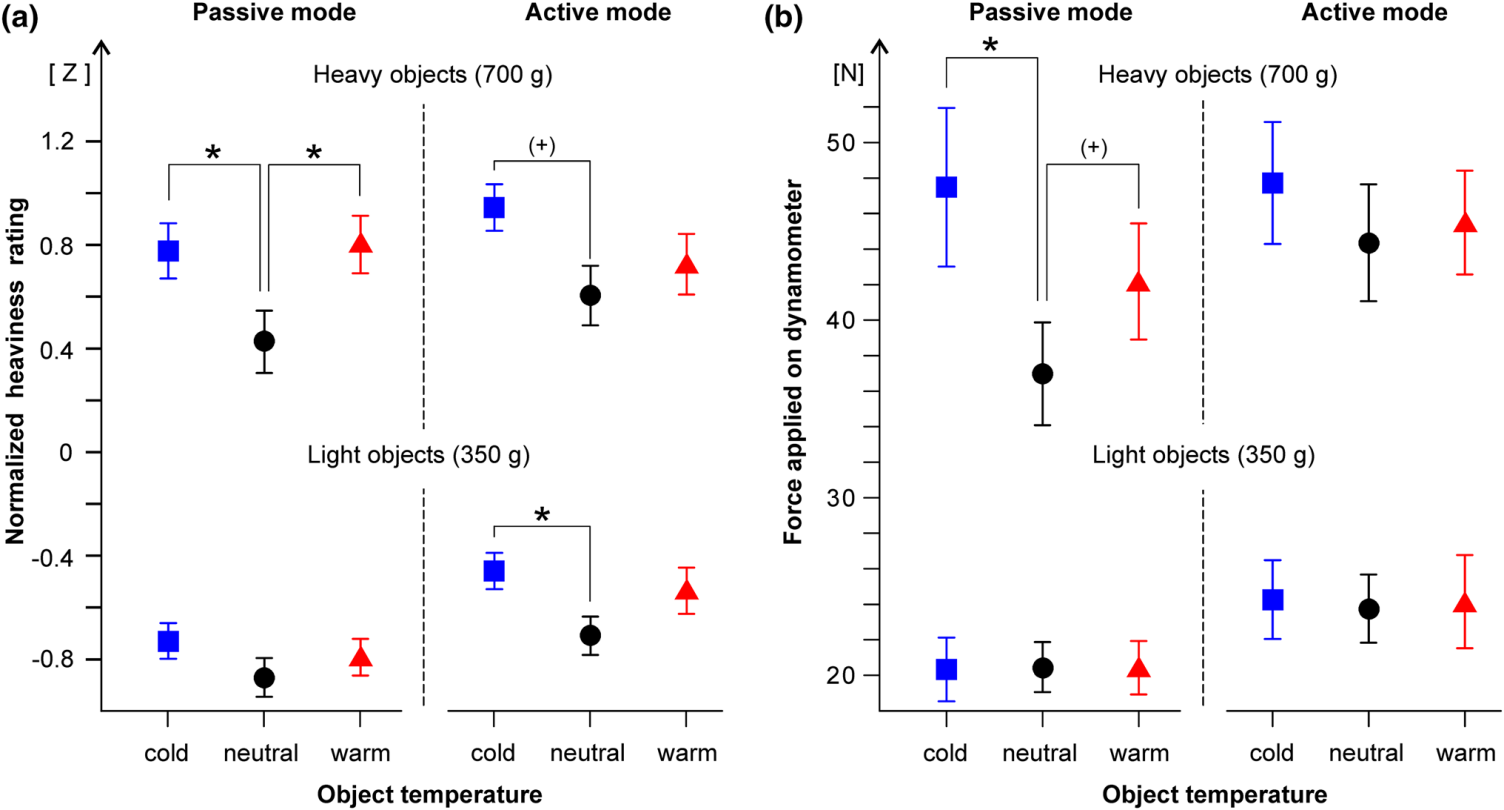
**a**, **b** Apparent heaviness. **a** Magnitude estimation: Symbols denote numerical ratings (interindividual mean ± standard error SEM) of the apparent heaviness of cold (blue squares), neutral (black dots) and warm (red triangles) test objects, which were placed onto the palm of the non-dominant hand. The numerical ratings have been standardized (z-transformation). Veridical object weight (350 g, 700 g) and mode of handling (active, passive) are indicated. **b** Cross modal matching: The mean height (± SEM) of the force pulses applied on the dynamometer as a measure of perceived heaviness is shown. Symbols and layout as in **a**). Significant effects (post-hoc *t* tests, corrected *p* < 0.05) of the temperature–weight illusion are marked with asterisks. Trends (corrected *p* < 0.1) are– indicated with ( +)

Computer-generated white noise was played in the background between the trials. At the onset of each trial the noise would diminish in loudness. On this cue, the experimenter placed one object onto the palm of the participant’s non-dominant hand, who focused on its heaviness during the following “attention” signal (400 Hz tone, 4.5 s long). A “go” signal (200 Hz tone, 4.5 s) prompted the subject to give a number representing the apparent heaviness. A short “stop” cue ended the trial. White noise followed. The object was removed, exchanged, and the next trial started after ~ 10 s. A designated value of “10” was assigned to the standard object (500 g, 32 °C) presented in the three initial calibration trials. Participants were blindfolded during subsequent trials, but held a standard weight (500 g) in their dominant hand as a constant reference. No constraints were placed except that larger numbers should represent heavier weights. The perceptual judgments were normalized into *z*-scores (subject-wise *z*-transformation), based on the mean and SD of each participant’s scores in the four runs (calibration trials excluded). Data were examined in a 2 (mode: active, passive) × 2 (actual weight: 350 g, 700 g) × 3 (object temperature: cold, neutral, warm) repeated-measures ANOVA. As in a related study (Buckingham et al. 2009), we describe effect sizes with partial squared eta (*η*^2^). Paired sample post-hoc *t* tests were used to compare the apparent heaviness of cold (warm) objects with neutral objects. We used one-tailed tests in line with the expected differences (cold > neutral, warm > neutral). Bonferroni corrections were applied to the eight post-hoc comparisons (see Fig. 3), so that *p* values of less than 0.0063 were considered statistically significant. Calibration and sham trials were not analyzed further.

#### Cross-modal matching

In their dominant hand (Fig. 1a), the participants held a strain-gauge-based isometric hand grip dynamometer (Noraxon® Clinical Dynamometer inline, MyoSystem 1400 L; Scottsdale, AZ, USA). Its analog output (± 5 V) was A/D converted at 1000 Hz (16-bit resolution) and was recorded and analyzed with an ADI Instruments Powerlab 8/30 system and LabChart 7 software (ADI Instruments, Oxford, UK). Auditory signals, stimulus presentation, and trial sequence were the same as during magnitude estimation (see above and Fig. 1b). Instead of giving a numerical value, however, the participant now squeezed the dynamometer during the “go” cue. The grip force should best match the perceived magnitude of the heaviness of the object that was placed on the non-dominant hand. The squeeze was released upon the “stop” cue. A designated force of 30 N was assigned to the standard object (500 g) presented in initial calibration trials. Here participants used visual feedback to align their force output with a 30 N target line shown on a monitor. The subsequent trials were performed without vision (monitor hidden behind curtains, participants blindfolded). Each trial consisted of one grip force pulse (see Fig. 1b). Pulse onset was defined when the force exceeded 10 N. We calculated the mean force over the central 2.5-s portion of each force pulse, excluding the initial rise (= first second after pulse onset) and the final rapid decline. The force data were entered into a 2 (mode) × 2 (weight) × 3 (temperature) repeated-measures ANOVA followed by post-hoc *t* tests as described above.

### Thermal sensitivity and skin temperature

The thenar eminence of the non-dominant hand was placed on a computer-controlled Peltier-type thermode (size of contact surface: 3 × 3 cm^2^) of a thermal sensory analyzer (model TSA 2001, Medoc Ltd., Israel). Starting from a baseline temperature of 32 °C, warm and cold detection thresholds were examined (three trials each) with ramp-like thermal stimuli (rate of change 1 °C/s). Participants signaled by button press when they detected the first change in temperature. Heat and cold pain thresholds were determined thereafter (three trials each, rate of change 1.5 °C/s). Volunteers were instructed to press the button at the first painful sensation. We emphasized that it was not the goal to examine pain tolerance. The mean thermal detection and pain thresholds were calculated from the respective trials. The protocol was the same as in a previous normative study of quantitative sensory tests (Rolke et al. 2006). We also measured the skin surface temperature at the thenar eminence with a contact thermometer (Testo Mini, Lenzkirch, Germany) and determined the peak grip force reached during a hard squeeze of the dynamometer and in each participant.

### Grip-lift experiment

Two Nano-17 force-torque sensors (ATI Industrial Automation, Garner, NC, USA) were fixed to a custom-built plastic and aluminum handle with opposing grip pads (Fig. 2a), which were covered with sandpaper (grit size 320). A bayonet lock allowed the handle and force sensors to be connected quickly to the current test object and removed easily after a trial (see supplementary Fig. 1). The force sensors and grip pads (but not the test objects) were at room temperature (~ 22 °C) in all trials. The cables of the sensors hung from a cantilever arm. The test objects’ actual weights, including handle and force sensors, were the same as in the psychophysical experiments (350 g, 700 g). Before the outset of the experiment, participants cleaned their hands to remove sweat and oil that may have reduced friction at the fingertips. Each subject performed four runs; the mode of handling (active, passive) and actual weight varied in a randomized order between runs. Each run included six sets of three consecutive grip-lifts of the same test object (Fig. 2b). Participants had full vision to allow for precise aim when grasping. Unlike in the prior psychophysical experiments, there were no calibration or sham trials.

Upon decreasing the loudness of white noise, an object (e.g., warm heavy) was set onto the palm of the subject’s non-dominant hand, where it stayed for ~ 4.5 s (auditory “attention” cue). In the passive mode, the non-dominant hand rested on the padded table (palm facing upward). In the active mode, the hand was held about 10 cm above the table (Fig. 2a). A tone with increasing pitch (500–1320 Hz, 1.5 s long) prompted the participant to grasp the handle between thumb and index finger of the dominant hand (precision grip) and to lift the object vertically in one smooth movement about 10 cm high (Fig. 2a). It was held there for ~ 3 s (“hold cue”, 1320 Hz). Upon a short “stop” cue (a ding), the object was placed on the table and released. White noise followed, and the next trial started ~ 7 s later (= experimenter took object, put it on participant’s hand → grip-lift-hold-release). After three trials, the test object was exchanged to start the next set of three with a different temperature (Fig. 2b). The actual object weight remained constant within each run to avoid aftereffects of changes in mass. The sequence of the temperatures within the runs was pseudorandomized (see supplementary Fig. 2).

Analog output (± 5 V) from the force sensors was digitized (sampling rate 1000 Hz) and processed with the same ADI Instruments (Oxford, UK) system as the dynamometer signal (see above). The grip force (GF) was the average of the forces of the thumb and index finger perpendicular to the grip surfaces. The lift force (LF) was the sum of the vertical forces exerted by both fingers (Fig. 2a). GF and LF curves were smoothed with a triangular Bartlett window (width 29 samples) and their first-order derivatives (force rates GFR, LFR) were calculated with a window width of 13 points. The maximum values of the forces (GF_max_, LF_max_) and the peak values of their rates of change (GFR_max_, LFR_max_) at the start of each grip-lift trial (Fig. 2b) were dependent measures (Johansson 1996). The timings of these maxima relative to the onset of the grip (defined as the instant when GFR exceeded 2 N/s), as well as the grip force GF_hold_ applied during static holding, 2 s after grip onset, were supplement variables.

Kinetic variables were examined in separate 2 (mode) × 2 (weight) × 3 (temperature) × 3 (trial) repeated-measures ANOVAs. The number of the trial within each set of three (1st, 2nd, 3rd) was included as an experimental factor, since it is known that expectation-driven scaling of the grip and lift forces is pronounced in the first trial. When illusion-inducing objects of identical weight are grasped and lifted repeatedly, the motor system rapidly adapts the force output to the objects’ actual mass (Buckingham et al. 2009; Buckingham and Goodale 2010). Post-hoc paired sample *t* tests (Bonferroni corrected) were used to assess the expected temperature-dependent effects on force output (one-tail tests: cold > neutral, warm > neutral) during the 1st and the 3rd trial.

## Results

### Perception of heaviness

Overall, identically weighted objects of the same appearance were perceived to weigh different amounts depending on their temperature (Fig. 3). Cold and, to a lesser extent, warm objects placed on the palm of the non-dominant hand appeared to be heavier than thermal-neutral objects. Object temperature had a significant main effect on the numerical ratings of apparent heaviness [*F*(2,40) = 9.59, *p* < 0.001, partial *η*^2^ = 0.32]. There were no significant interactions with the actual weight or mode of handling (active, passive). When placed onto the palm of the resting hand (passive mode), warm and cold 700 g objects felt significantly heavier (Fig. 3a) than neutral objects of the same weight (post-hoc tests, corrected *p* < 0.05). When the objects were carried actively, an analogous significant difference (corrected *p* = 0.045) was found for cold light objects (350 g) and a trend (corrected *p* = 0.1) for cold heavy objects. Unsurprisingly, the veridical mass (350 g vs. 700 g) had a significant main effect on the perceived heaviness [*F*(1,20) = 359.1, *p* < 0.001, partial *η*^2^ = 0.95]. The influence of the mode of handling (active, passive) did not reach significance [*F*(1,20) = 3.8, *p* = 0.065, partial *η*^2^ = 0.16].

During cross-modality matching (Fig. 3b), the blindfolded participants expressed the magnitude of the objects’ apparent heaviness by squeezing a dynamometer with the dominant hand. Object temperature had a significant effect on the squeeze force [*F*(2,40) = 4.99, *p* = 0.012, partial *η*^2^ = 0.20], with a significant interaction between actual weight and temperature [*F*(2,40) = 4.71, *p* = 0.015, partial *η*^2^ = 0.19]. Post-hoc tests showed that the cold 700 g objects appeared to be significantly heavier (corrected *p* = 0.03) than neutral objects of the same weight (Fig. 3b). A similar trend (corrected *p* = 0.08) was found for warm 700 g objects in the passive mode of handling. Compared to the mean squeezing force of 31.37 N that described the apparent heaviness of neutral objects, the force applied on the dynamometer was 11% higher (34.93 N) when cold objects were judged, and 5% higher (33.05 N) when the objects were warm. As was expected, the influence of the actual weight (350 g, 700 g) was significant [*F*(1,20) = 77.11, *p* < 0.001, partial *η*^2^ = 0.79]. Also the mode of handling had a significant main effect during cross-modality matching [*F*(1,20) = 10.34, *p* = 0.004, partial *η*^2^ = 0.34]. The mean force applied on the dynamometer (averaged across the other conditions) was lower in the passive (31.3 N) than in the active mode (34.9 N) of handling so that the objects were judged to be heavier when they were carried actively. Numerical ratings of apparent heaviness (Fig. 3a) and cross-modality matching (Fig. 3b) yielded largely congruent results. Significant positive correlations between data obtained with both methods were found for perceptual ratings of the 700 g object’s heaviness (see supplementary data).

### Sensory testing

The average warm detection threshold at the thenar eminence was 33.4 ± 0.13 °C (mean ± standard error) and the cold detection threshold 30.3 ± 0.12 °C in the tests where the baseline thermode temperature was set at 32 °C. The heat pain threshold was 43.6 ± 0.71 °C, and cold pain began at a mean temperature of 14.1 ± 1.5 °C. The results fall within the range of published normal data obtained with the same protocol (Kuhtz-Buschbeck et al. 2010; Rolke et al. 2006). None of the participants reported painful sensations when handling the warm (41 °C) and cold (18 °C) test objects. The mean skin temperature at the thenar eminence was 30.3 ± 0.32 °C and, therefore, somewhat lower than the neutral temperature (32 °C). In one condition (active mode, 700 g object, 32 °C) of the cross-modality matching experiment there was a significant negative correlation (Pearson’s *r* = − 0.48, *p* < 0.05) between individual skin temperatures and perceptual ratings of heaviness. Such correlations were found neither in the other eleven conditions nor in the grip-lift experiments. A peak force of 237 ± 16 N was reached during a hard squeeze of the dynamometer. Hence the forces applied during cross-modal matching (Fig. 3b) stayed well below 25% of the maximum force and did not cause fatigue.

### Grip-lift experiment

**Fig. 4:**
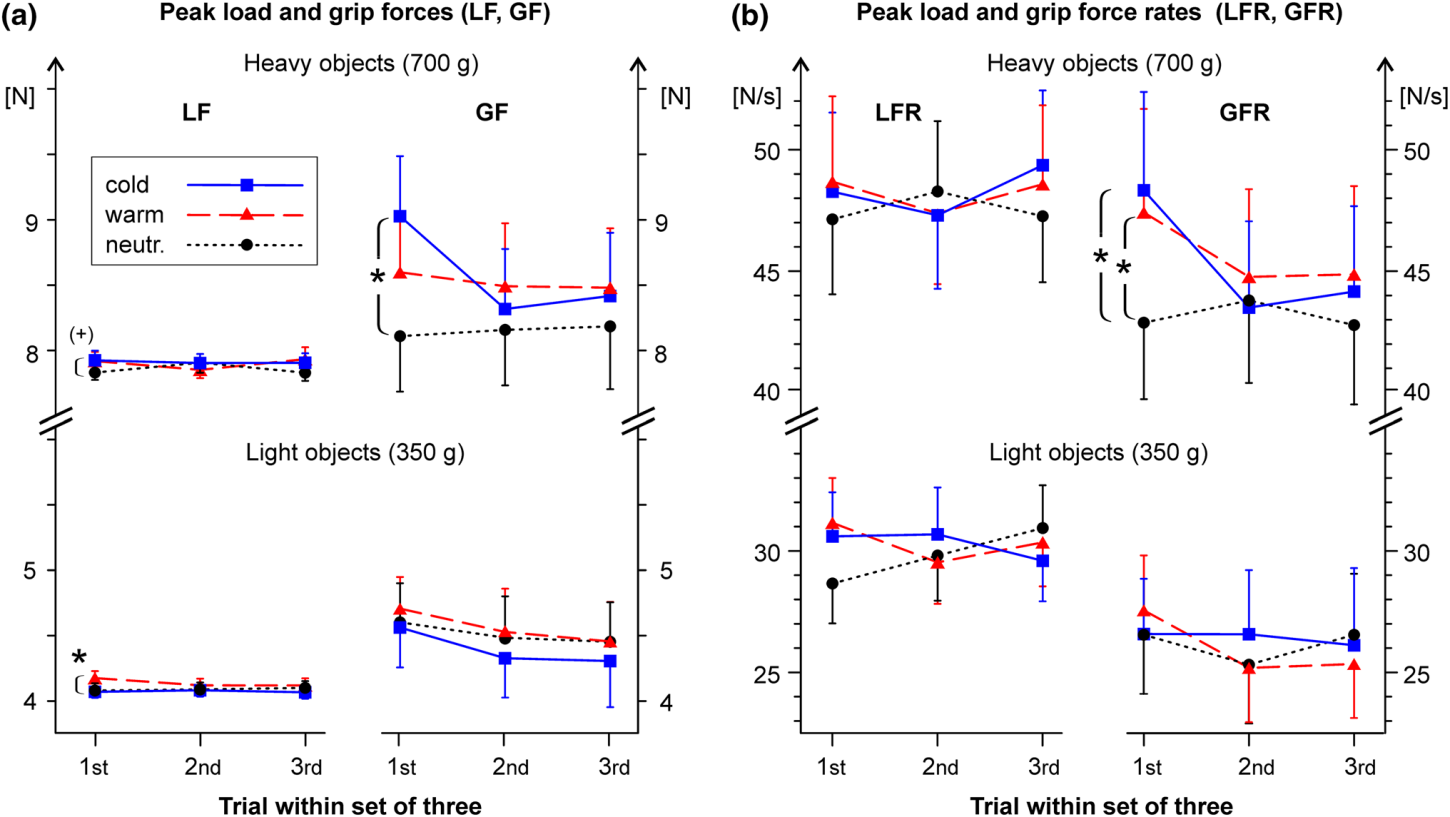
**a**, **b** Grip-lift task results. **a** Peak lift (LF) and grip (GF) forces applied to heavy (700 g) and light (350 g) test objects of different temperatures (18, 32, 41 °C) during the first, second and third trial during the sets of three consecutive grip-lift trials (see Fig. 2b). Data indicate inter-individual mean values ± standard error (SEM), averaged across both modes of handling (active, passive). Symbols as in Fig. 3. **b** Peak lift force rates (LFR) and grip force rates (GFR). Significant effects (post-hoc *t* tests, corrected *p* < 0.05) of the temperature–weight illusion are indicated with asterisks. A trend (corrected *p* = 0.1) is marked with ( +)

Unsurprisingly, the actual object mass had strong significant effects (*p* < 0.001) on LF_max_ [*F*(1,20) = 12,220, partial *η*^2^ = 0.99] and GF_max_ [*F*(1,20) = 411.9, partial *η*^2^ = 0.95], as well as on the peak force rates LFR_max_ [*F* (1,20) = 151.9, partial *η*^2^ = 0.88] and GFR_max_ [F(1,20) = 154.7, partial *η*^2^ = 0.89]. The trial number (1st, 2nd, or 3rd within a set of three) had a significant main effect on GFR_max_ [*F*(2,40) = 5.37, *p* = 0.009, partial *η*^2^ = 0.21], whereas the mode of handling (active, passive) had no significant influence on force variables. Figure 4, therefore, shows data that have been averaged across the two modes.

In line with our hypothesis, the temperature of the test objects had significant effects on LF_max_, GF_max_, and GFR_max_. In terms of LF_max_, we found a main effect of temperature [*F*(2,40) = 3.70, *p* = 0.034, partial *η*^2^ = 0.16], and a significant interaction between trial number and temperature [*F*(4,80) = 2.91, *p* = 0.026, partial *η*^2^ = 0.13]. Post-hoc testing showed that in the 1st trial—but not in the 3^rd^—higher LF_max_ was applied to warm light objects (corrected *p* = 0.02) as compared to neutral light objects (Fig. 4a). A similar trend towards an increased LF_max_ was found for cold heavy objects (corrected *p* = 0.1). The differences (cold minus neutral) in LF_max_ were small in absolute (< 0.1 N) and in relative (+ 1.5%) terms. The GF_max_ measure showed a main effect of the temperature [*F*(2,40) = 4.34, *p* = 0.02, partial *η*^2^ = 0.18], with a significant interaction between temperature and veridical object weight [*F*(2,40) = 6.79, *p* = 0.003, partial *η*^2^ = 0.25]. Post-hoc testing showed that participants applied a significantly higher GF_max_ (corrected *p* = 0.014) to cold heavy objects than to otherwise identical neutral objects at the first trial (Fig. 4a). The corresponding difference was ~ 0.9 N (+ 10% in relative terms). The GFR_max_ yielded a main effect of temperature [*F*(2,40) = 3.43, *p* = 0.04, partial *η*^2^ = 0.15] without significant interactions (Fig. 4b). On average, about 4% higher rates of grip force were applied to cold (mean: 35.9 N/s) and warm (35.8 N/s) objects than to neutral objects (34.6 N/s). Post-hoc tests showed that GFR_max_ was significantly increased above the “neutral baseline” during lifts of cold heavy objects (corrected *p* = 0.02) and warm heavy objects (corrected *p* = 0.05) during the 1st trial (Fig. 4b). All in all, the temperature–weight illusion led to clearly elevated grip forces and force rates (GF_max_, GFR_max_) when cold heavy objects (700 g) were grasped and lifted for the first time during a set of three consecutive trials. The objects were grasped in a way that suggests a perceived illusory increase of their mass by 5–10%. When warm heavy objects were grasped, the grip force rate GFR_max_, but not the peak force (GF_max_), was significantly increased. The additional variables (GF_hold_, time variables) yielded no significant main effects of temperature (see supplementary material). Anonymized individual results of the psychophysical and the grip-lift experiments are available as a supplementary data file.

At the very end of the experiments, a tepid (32 °C), and a cold (18 °C) coin of the same kind were placed sequentially on the forehead of each participant (head reclined). Nineteen (of twenty-one) participants said without hesitation that the cold coin felt heavier, thus confirming Weber’s silver thaler illusion (Ross and Murray 1996), which none of them had known before.

## Discussion

In the nineteenth century, Weber noted that a cold coin placed on the forehead of a supine person feels heavier than a warmer coin. This temperature–weight illusion (TWI) was reinvestigated by Stevens and colleagues (Stevens and Green 1978; Stevens 1979), who placed cold (0 °C), neutral (32 °C), and warm (45 °C) aluminum disks on the skin of healthy volunteers and let them rate the perceived heaviness. Unfortunately, the cold and warm temperatures were in the noxious range (Rolke et al. 2006) so that discomfort and pain may have biased the results. Effects of the TWI on a grip-lift task have not been investigated up to now. If cold (or warm) objects are expected to be heavier than thermal-neutral objects of the same shape, material and size, the motor system will predictively increase the grip and load forces applied by the fingers when such items are grasped and lifted. In the present study, we could elicit a TWI with innocuous thermal stimuli, namely with cold (18 °C), warm (41 °C), and thermal-neutral (32 °C) objects that were placed on the palm of the non-dominant hand. Cold and warm objects felt heavier than neutral, identically weighted objects. In line with the higher apparent heaviness, subjects applied increased grip force when grasping cold objects with the dominant hand to lift them off the palm of the other hand in initial grip-lift trials. The TWI was stronger with cold than with warm objects and stronger with heavy than with light objects. Proprioceptive sensory information about the actual weight of the objects (active mode) did not completely eliminate the TWI.

### Neurophysiology

As one explanation for the TWI, Stevens and Green (1978) argued that mechanoreceptors that respond to skin indentation also react, albeit less sensitively, to thermal stimulation. Microneurographic studies found some cold sensitivity in large myelinated afferent fibers supplying slowly adapting (SA) mechanoreceptors (Schepers and Ringkamp 2010). These receptors respond primarily to pressure and shearing of the skin, but when additional cooling increases their discharge rates (Schepers and Ringkamp 2010), this may elicit a TWI. In agreement, a cooled pressure stimulus applied on the dorsum of the hand is perceived as “heavier” than a neutral stimulus, and a compression block of the myelinated fibers supplying this region was found to reduce the incidence of this TWI (Dunn et al. 2017). However, since the compression block did not completely eliminate the illusion, thermal sensitivity of unmyelinated mechanosensitive fibers might also contribute to the TWI. Concerning molecular mechanisms, TRPM8 channel agonists consistently enhanced the cold responsiveness of slowly adapting mechanoreceptors in a pharmacological study (Cahusac and Noyce 2007). Some of these mechanoreceptors also increased their activity transiently at temperatures between 42 and 45 °C (see Fig. 2c of that study).

### Thermal clues to the material

Cold and to a lesser extent, warm objects might be associated with materials whose high density is approximately known (Ho and Jones 2006). Metal generally feels colder to the touch than plastic and wood, and metal is heavier. The contact coefficient represents a material’s ability to conduct and store heat (Ho 2018). Aluminum and other metals have more than tenfold higher contact coefficients than wood, plastic and foam (Ho 2018, her Fig. 3). Metal at room temperature feels cold because it elicits a high initial cooling rate and a large total change in skin temperature. Likewise, hot metal warms the skin rapidly, so that it feels burning hot in a sauna whereas wood does not. Possibly the participants associated the cold and warm test objects with metal as a dense heavy material and, therefore, applied higher forces in the initial grip-lift trials. The coldness-induced increase of GF_max_ was about + 10%, while LF_max_ increased by about + 1.5%, compared to lifts of the identically weighted neutral object (Fig. 4). Since the participants could see the objects to allow for targeted grasping, a discrepancy between the association of coldness with heavy metal and the visible surface material (plastic and aluminum) of the objects may have arisen and attenuated the effect of the temperature.

### Perception of illusory moisture

The perception of moisture in the context of cooling the skin may be relevant to the present experiments. Cutaneous cold afferents and tactile afferents (low level of mechanical pressure) play a key role in the ability to sense skin wetness (Filingeri and Havenith 2015). In a recent study (Carnahan et al. 2010), eight volunteers grasped and lifted objects (mass 400 g) whose brass grasping surfaces were either cold (16 °C) or at room temperature (24 °C). The grip force during static holding was about 25% higher when the when the surfaces were cold. A perception of illusory wetness and slipperiness associated with the cold grasping surfaces accounted for the higher grip force found in that study, and indeed the participants rated the cold object as being wetter than the warmer object. Cold-dry stimuli applied to the forearm are known to induce the illusion of skin wetness, too (Filingeri et al. 2013). Warm-wet and neutral-wet stimuli applied to forearm and index finger pad are perceived as less wet than cold-wet stimuli (Filingeri et al. 2014). However, unlike in the experiment of Carnahan et al. (2010), the grasping surfaces of the force transducers were always at room temperature and dry during our grip-lift task. Only the attached cylinders with the aluminum base plates were cooled or warmed (see supplementary Fig. 1). Although cold objects may per se be associated with wetness and slipperiness, the perception of illusory moisture at the grasping surfaces did not play a role in the present study.

### Actual weight of test objects

We used 350 g and 700 g objects to elicit and test the illusion and found significant interactions between the actual weight and the objects’ temperature during cross-modal matching (dynamometer) and for GF_max_ in the grip-lift task (see Figs. 3b and 4). Here heavy cold objects induced a significant TWI, whereas light cold objects did not. The thermal contact resistance decreases and subcutaneous blood vessels are compressed when the contact force is higher so that the skin temperature changes more rapidly when heavy objects are placed on the palm (Galie and Jones 2010; Ho 2018). In daily experience, the sensation of cooling (or warming) is more intense when the hand is in tighter contact with an object. Nevertheless, Stevens (1979) found that light cold aluminum disks (21 g) placed on the skin induced a stronger TWI than heavy (105 g) cold disks did. He conjectured that the cooling of the skin adds a constant increment in the activity of skin nerves. Since this increment is higher in proportion to the baseline activity when the pressure is light, the relative degree of cold intensification may be higher for light than for heavy objects. However, this phenomenon was not obvious at the palm, but only at other body sites [see convergence of lines in Steven’s (1979) Fig. 1].

### Body region

We placed the test objects onto the palm of the hand, but the TWI may be stronger at other body sites. Stevens (1979) found that cold intensification of pressure stimuli was relatively weak at the palm: when placed on the volar forearm, the apparent heaviness of a cold light metal disk (0 °C, 21 g) was two times higher than the heaviness of a neutral disk of the same weight, whereas this factor was only about 1.1 at the palm (Table 1 in Stevens 1979). Regional differences in tissue stiffness might be relevant. The skin and subcutaneous tissue of the volar forearm are softer than the palm of a flat hand (Moore and Mundie 1972). A given local pressure will, therefore, cause stronger skin deformation and possibly more activation of cutaneous mechanoreceptors, whose activity is boosted by additional coldness. With stimuli of 0 °C (Stevens 1979), cold pain and habituation may play a role. It is more common to touch cold and warm objects with the hand than with other body sites, so thermal sensations may be less vivid and as a consequence, the TWI weaker at the palm. Finally, signals from receptors of the hairy skin may be processed differently from receptors in the glabrous skin of the hand, which typically contribute to tactile identification of objects (Galie and Jones 2010).

### Proprioceptive sensory information

Only thermal and tactile cues (pressure, skin deformation) to heaviness were present in the passive mode, when the object was placed onto the non-dominant hand that rested on the table (palm facing upward). Additional proprioceptive information became available in the active mode, when subjects carried the test object on their non-dominant hand, even without moving it. These proprioceptive cues (active force production, muscle tension) gave unbiased information about the objects’ weight. In congruence, the TWI was somewhat stronger in the passive than in the active mode in the cross-modality matching experiment, but the proprioceptive information available in the active mode did not completely abolish the illusion (Fig. 3). Active holding generally increased the apparent heaviness (active > passive) during this experiment, possibly reflecting the effort needed to carry the object (Proske and Allen 2019). Weight is sensed most precisely when an object is grasped and lifted under dynamic conditions (Brodie and Ross 1984; Jones 1986). In the present grip-lift experiments, the non-dominant hand held a cold (resp. warm) seemingly heavier object, but the force needed to lift it with the dominant hand already provided information about its actual mass in the first trial. Therefore, the LF and GF could be scaled appropriately in the subsequent trials.

### Related weight illusions

The material–weight illusion (MWI) and the size–weight illusion (SWI) are likely to result from a discrepancy between prior expectations and sensory information about object weight (Buckingham 2014). A MWI is induced by variations in the surface material of identically weighted objects of the same size that are grasped and lifted. Since metal is expected to be heavier than, e.g. polystyrene, a heavy-looking object with a metal surface is grasped and picked up with more force than a lighter-looking object in initial grip-lift trials (like the cold object was in the present study). But since, unexpectedly, the heavy-looking object weighs as much as the lighter-looking object, the former is perceived as lighter than the latter (MWI). This illusion persists over many grip-lift trials even after the motor system has scaled the grip and lift forces to the objects’ actual mass (Buckingham et al. 2009, 2011). Similar data were reported for the size–weight-illusion, where a large object consistently felt lighter than an identically weighted small object (Buckingham and Goodale 2010). To examine the TWI in an analogous way, identically weighted cold and thermal-neutral objects would need to be first touched and then grasped and lifted in a series of trials. After each lift, participants would be asked to rate how heavy the object felt. If there was a strong expectation that cold objects are normally heavier than thermal-neutral objects, the violation of this expectation could induce the illusion that the cold items are comparatively light. On the other hand, the cold objects feel heavier when they are placed onto the skin (as shown in the present study). A study of this discrepancy would be an interesting topic for future research.

The present study has limitations. Only two different actual object weights and three temperatures were studied. The size of the skin area exposed to the thermal stimuli was not varied. Further research may include a greater range of stimulus levels to determine a possible progression in the effects of temperature on the perceived heaviness of objects. In the present study, we assumed a neutral baseline temperature of 32 °C in line with sensory testing protocols (Rolke et al. 2006) and previous research (Stevens 1979), but this baseline did not exactly match the actual skin temperature of the participants’ hands (mean value: 30.3 °C). Skin temperature in the extremities can vary by 5 °C across different subjects, and sensory thermoneutrality is possible outside the 32–34 °C range (Filingeri et al. 2017). It would have been better to adapt the neutral object temperature to the individual skin temperature. Further research could examine how changes of the skin temperature influence the TWI.

In conclusion, a temperature–weight illusion (TWI) was elicited with innocuous thermal stimuli (18, 32, 41 °C) in the present study. The TWI influenced both perception of apparent heaviness, as well as action, namely the anticipatory scaling of the grip force. This force was increased when cold objects were grasped and lifted from the palm of the hand, compared to lifts of thermal-neutral objects of identical weight. The TWI was less strong with warm objects.

## Electronic supplementary material

Below is the link to the electronic supplementary material.

Supplementary file1 (PDF 570 kb)

Supplementary file2 (XLSX 240 kb)
