# Supplementary material for: Cold and heavy: grasping the temperature–weight illusion
Source: Exp Brain Res. 2020 Mar 27;238(5):1107–17. doi: 10.1007/s00221-020-05794-y (PMC7237526; doi:10.1007/s00221-020-05794-y)
Supplement: Supplementary file 1 — Supplementary file1 (PDF 570 kb) [file 221_2020_5794_MOESM1_ESM.pdf]

SUPPLEMENTARY MATERIAL
for article "Cold and heavy: grasping the temperature-weight illusion"
by Kuhtz-Buschbeck and Hagenkamp

A supplementary file (Excel-spreadsheet "TWI supplementary data.xls") with anonymized results of the 21 participants is provided separately. It includes explanations, scatterplots with individual datapoints, and correlations between the results of the two psychophysical experiments (magnitude estimation, cross-modal matching).

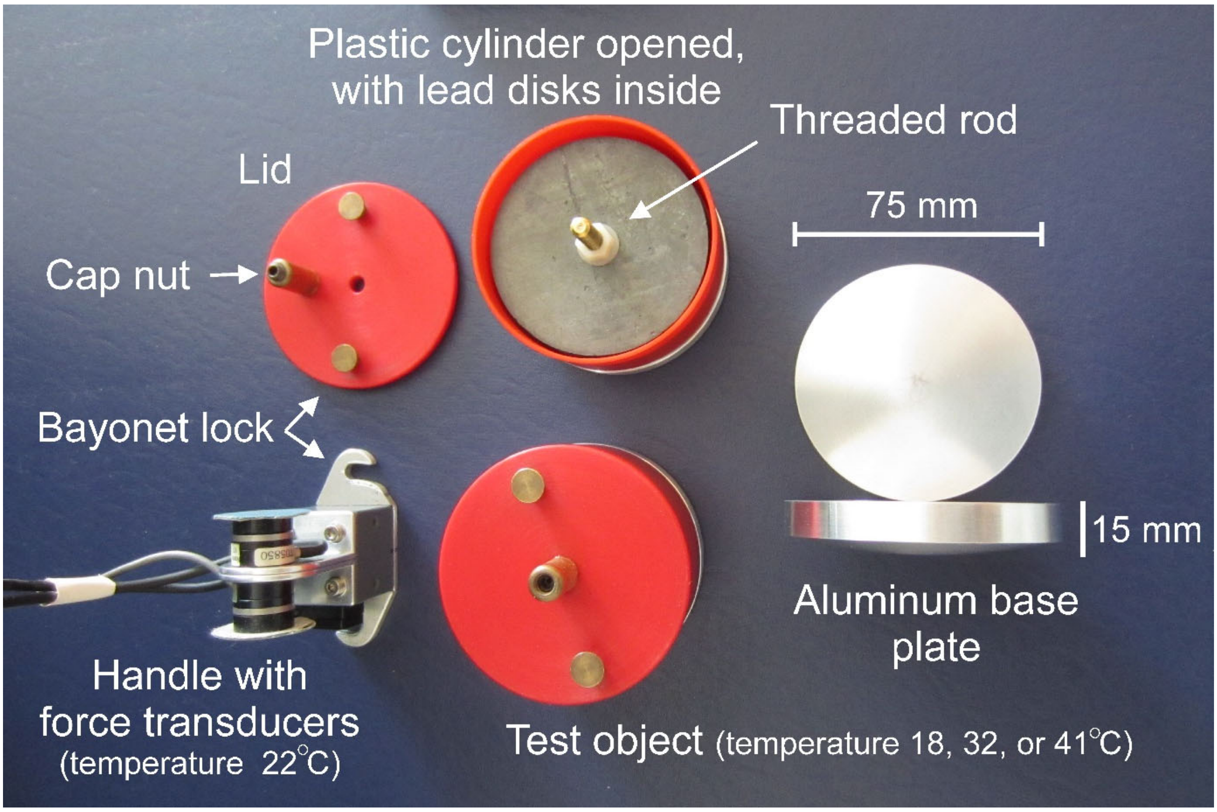

Supplementary Figure 1: Test objects.

Objects were red hollow plastic cylinders with a massive (15 mm thick) aluminum base plate, the bottom of which was slightly curved so that it fit onto the palm of the flat hand. A custom-made handle with force transducers was attached to the object with a bayonet lock before each grip-lift trial. The plastic cylinders were filled with lead disks, which were fastened to a threaded rod. The test objects were warmed or cooled to the desired temperatures (41°C, 32°C, 18°C) with a warming cabinet and cool boxes.

The handle, force transducers and grip surfaces were at room temperature (ca. 22°C) in all trials.

The total weight of the objects (including cylinder, lead, baseplate, attached handle with tranducers etc.) was either 350 g (light) or 700 g (heavy).

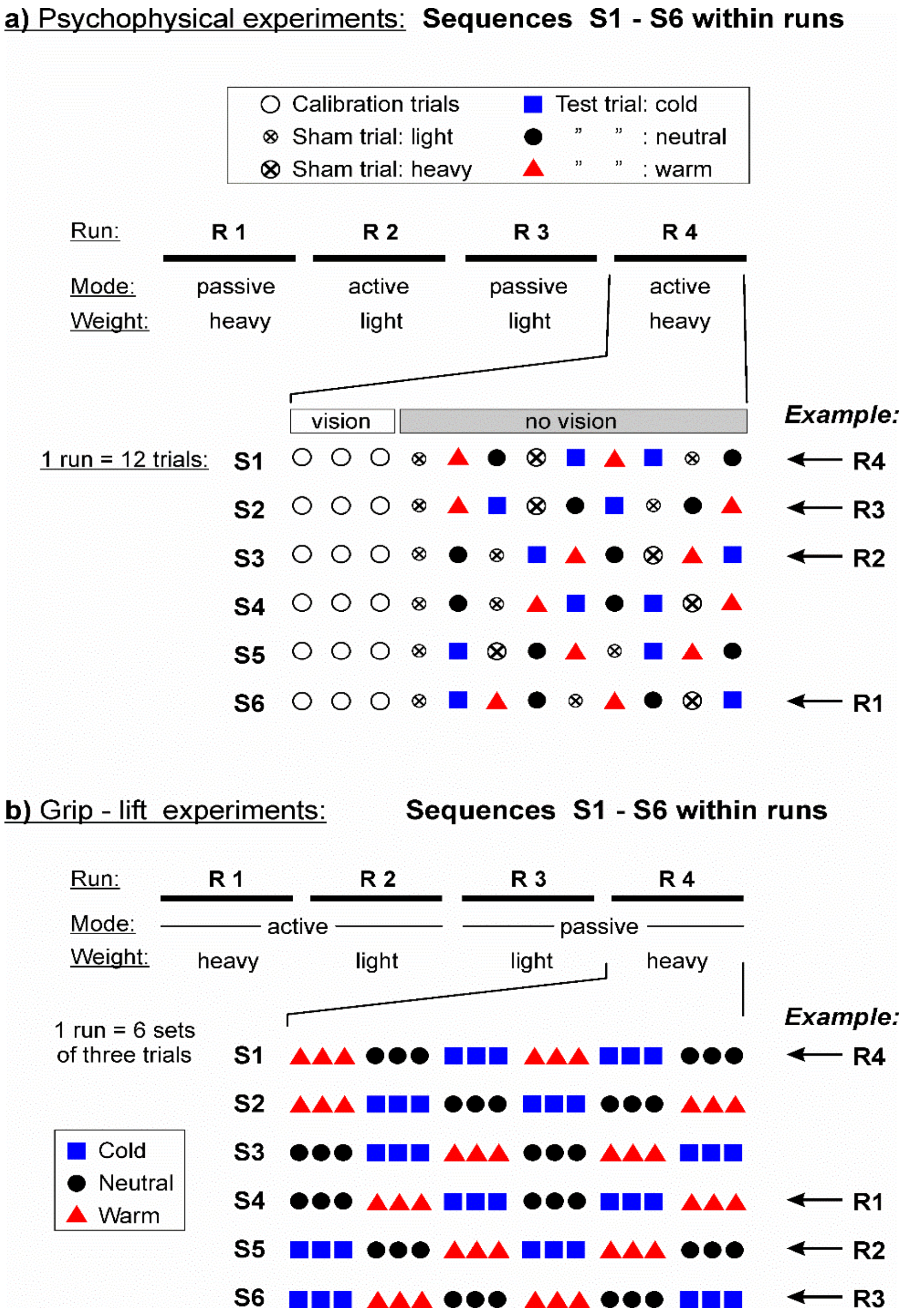

Supplementary Figure 2: **Pseudorandomized order of conditions.**

a) Psychophysical experiments. In each participant, four out of six possible pseudorandomized sequences S1-S6 were used for experimental runs R1 to R4 (see example). Mode of handling (active or passive) and veridical object weight (350 g or 700 g) remained constant within each run. Following three initial calibration trials (with 500 g standard weight), test objects were presented. The same temperature was never tested twice in succession during a sequence. Interspersed sham trials (with light and heavy dummy objects) never occurred twice in succession either. Across all experiments, the six possible temperature sequences (warm→cold, warm→neutral, cold→warm, cold→neutral, neutral→cold, neutral→warm) occurred with equal frequency. Moreover, test objects of each temperature were preceded by sham trials (light dummy, heavy dummy) with equal frequency; e.g., light dummy→cold test object, light dummy→warm test object, light dummy→neutral test object were equally frequent.

b) Grip-lift experiments. No calibration or sham trials were performed here. The order of the temperatures (lifted in bouts of 3 trials) and conditions was pseudorandomized as outlined above.

**Grip-lift variables**	Cold objects (18 °C)	Neutral obj. (32 °C)	Warm objects (41 °C)	Effect of temperature (ANOVA)
GF max [N]	6.62 ± 0.37	6.45 ± 0.36	6.67 ± 0.36	$F_{2,40} = 4.3$ *
GFR max [N/s]	36.57 ± 3.07	35.32 ± 2.81	36.55 ± 2.97	$F_{2,40} = 3.4$ *
LF max [N]	6.11 ± 0.06	6.09 ± 0.06	6.13 ± 0.06	$F_{2,40} = 3.7$ *
LFR max [N/s]	40.06 ± 2.38	39.43 ± 2.23	40.04 ± 2.42	$F_{2,40} = 0.7$
Time of GF max [ms]	397 ± 20	405 ± 19	405 ± 20	$F_{2,40} = 2.0$
Time of GFR max [ms]	155 ± 11	160 ± 11	159 ± 12	$F_{2,40} = 1.2$
Time of LF max [ms]	391 ± 22	392 ± 21	381 ± 20	$F_{2,40} = 2.0$
Time of LFR max [ms]	187 ± 10	190 ± 11	187 ± 10	$F_{2,40} = 0.4$
GF hold [N]	5.43 ± 0.31	5.37 ± 0.29	5.43 ± 0.30	$F_{2,40} = 0.8$

Supplementary Table: Grip-lift measures

Mean results of grip and lift force measures (± standard error of the mean) during grip-lift trials with cold, neutral and warm test objects. The data have been averaged across the other conditions, i.e. across mode (active, passive), weight (heavy, light) and trial number (1st, 2nd, 3rd). ANOVA – F values describing effects of the temperature are shown in the right column. Main effects of temperature were significant (* $p < 0.05$) for GF max, GFR max, LF max.

Abbreviations: GF max, peak grip force; GFR max, peak grip force rate; LF max, peak lift force; LFR max, peak lift force rate; Time of GF max etc., timing of these maxima relative to grip onset; GF hold, grip force two seconds after grip onset, when the lifted object was held statically. Grip onset was defined as the moment when GFR exceeded 2 N/s (see Methods).
